# A case report of branch retinal artery occlusion in a teenager due to hyperhomocysteinaemia; the interplay of genetic and nutritional defects

**DOI:** 10.1186/s12886-018-0859-2

**Published:** 2018-09-14

**Authors:** Clare Shute

**Affiliations:** Royal Victoria Hospital, Belfast, Northern Ireland

**Keywords:** Branch retinal artery occlusion, Homocysteine, Hyperhomocysteinaemia, MTHFR, Vitamin B12, Folic acid

## Abstract

**Background:**

Retinal vascular occlusions are uncommon in young people and require more in-depth investigation into the cause. Studies have revealed that a high level of circulating homocysteine poses a risk for retinal vaso-occlusive events across a wide age range. This case report reflects on how the interplay of genetic mutation and vitamin deficiency can cause a pathological level of homocysteine with resultant branch retinal artery occlusion in a young patient.

**Case presentation:**

A 16-year-old boy presented to eye casualty with acute inferior visual field loss in the left eye. Visual acuity remained normal at 6/6 each eye and the event was painless. Initial assessment, and retinal photography revealed a left superior hemi-field branch retinal artery occlusion with macular sparing. Given the patient’s age, extensive investigation into the cause was carried out. Positive findings were of an elevated level of homocysteine as a result of vitamin B12 and folic acid deficiency as well as a genetic mutation in the MTHFR gene (encoding MTHFR enzyme which is vital in normal homocysteine metabolism). Vitamin B12 and folic acid were replaced which in turn normalized the patient’s homocysteine levels. At two months, the patient’s visual fields had also improved, and no further vascular event had occurred.

**Conclusions:**

This case report has highlighted the link between hyperhomocysteinaemia and retinal artery occlusion. However, despite vitamin replacement being shown to normalize homocysteine levels, no evidence exists to date as to whether this will reduce the risk of further retinal vascular occlusion.

## Background

Retinal vascular occlusions are rare in the teenage population and a more extensive investigation into the cause is required given that a thrombophilia or autoimmune disorder may present in this manner. Homocysteine levels are one of the recommended investigations in young patients as hyperhomocysteinaemia is an increasingly recognized cause for both retinal artery and vein occlusions [1–3]. Studies have shown that 5–10% of the general population have varying levels of hyperhomocysteinaemia [4] and this can reach to 30–40% in the elderly [5]. High homocysteine levels cause accelerated vascular atherosclerosis by encouraging thrombosis, oxidant stress, endothelial cell damage and vascular smooth muscle proliferation [6, 7]. As a result, hyperhomocysteinaemia has been shown to be an independent risk factor for vascular events including myocardial infarction, cerebrovascular events and retinal vaso-occlusive disease [8–12]. A knowledge of homocysteine metabolism aids understanding as to the cause of hyperhomocysteinaemeia with genetic defects in vital enzymes as well as deficiencies of important vitamin cofactors being implicated [6].

Replacement of vitamin cofactors involved in homocysteine metabolism has been shown to normalise homocysteine levels [13]. However, meta-analysis of research articles evaluating vitamin replacement for hyperhomocysteinaemia has failed to show that resultant reduction of homocysteine levels can reduce the risk of major vaso-occlusive events [13]. It is also important to note that, although the relationship between hyperhomocysteinaemia and retinal vascular occlusion has been increasingly reported [14–19], there are is no current research into whether normalising homocysteine levels results in reduction in risk of further retinal vaso-occlusive events [12, 14].

This case report supports existing literature in revealing how the interplay of genetic and nutritional defects can cause a pathological level of homocysteine with resultant vascular occlusion.

## Case presentation

A 16-year-old boy presented to eye casualty having noticed a brief episode of flashing lights followed by acute inferior hemi-field visual loss in the left eye while walking to class. The visual loss persisted, and the event was completely painless. Past medical history consisted only of migraine which the patient was not experiencing at the time of visual field loss. He denied any history of smoking, illicit drug use, alcohol consumption or sexually transmitted infections.

**Fig. 1:**
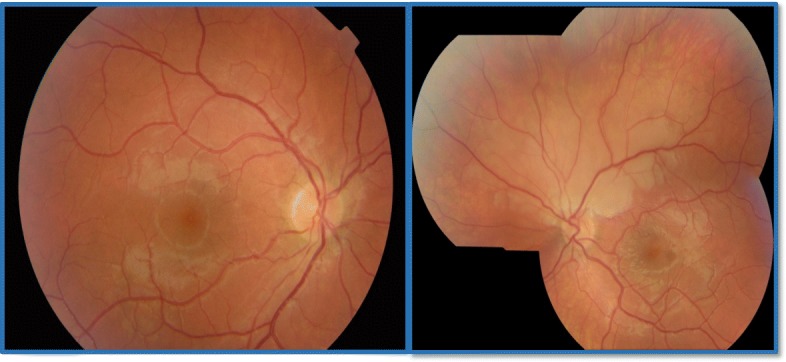
Colour retinal photographs revealing retinal pallor and arterial attenuation across superior retinal arcade in the left eye (Right eye –normal)

**Fig. 2:**
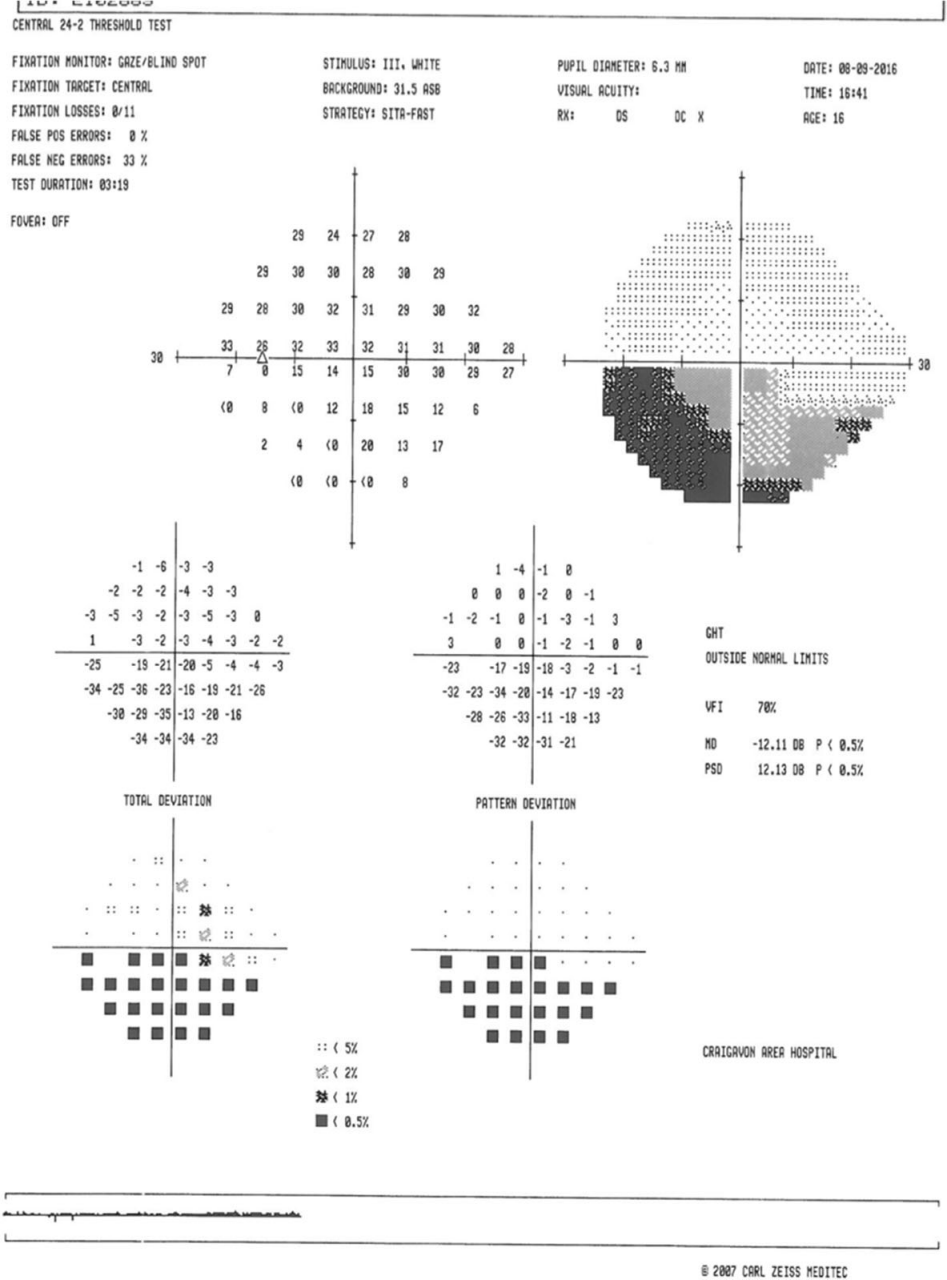
Visual field test of the left eye 2 days post presentation. An inferior altitudinal defect is demonstrated

**Fig. 3:**
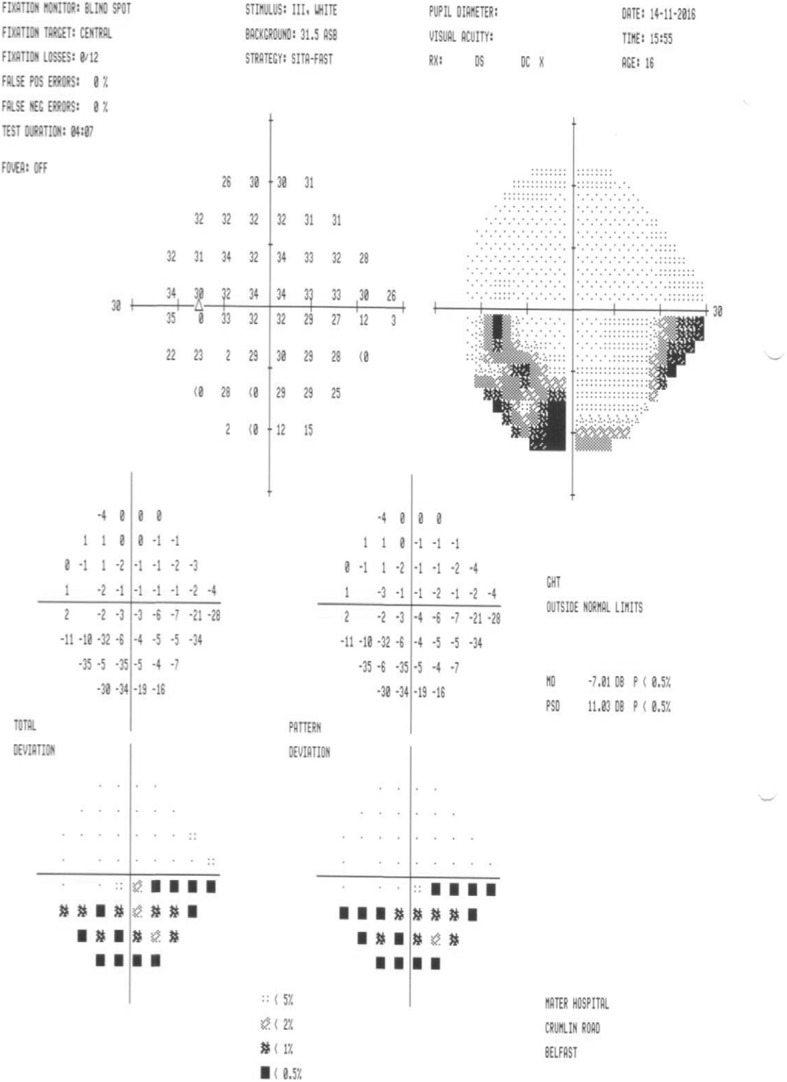
Visual field test of the left eye 2 months post presentation. Moderate improvement in the inferior altitudinal visual field defect is demonstrated

Examination revealed a normal visual acuity of 6/6 in both eyes. A clear inferior altitudinal defect was evident when visual fields were tested to confrontation and a supra-temporal wedge of retinal pallor with associated arterial attenuation was visualized on slit lamp ophthalmoscopy. Colour retinal photography confirmed evidence of a superior branch retinal artery occlusion (BRAO) (ref. Fig. 1) and visual field testing confirmed an inferior altitudinal field defect in the left eye (ref. Fig. 2). Aspirin was commenced at this point and the patient was referred for urgent review with the local stroke team. Trans-thoracic echocardiography revealed a bicuspid aortic valve but reassuringly no vegetations that may have produced emboli. MRI and MR-angiogram of brain and neck were unremarkable. Haematological investigations revealed a moderate-severely raised homocysteine level (68.0 μmol/L, normal range 5.5–13.6 μmol/L), vitamin B12 deficiency (108 ng/L, normal range 191–663 ng/L) and a borderline folate deficiency (4.6 μg/L, normal range 4.6–18.7 μg/L). Following these findings, genetic testing was completed, revealing a C677T subtype homozygous mutation for the gene encoding methylenetetrahydrofolate reductase (MTHFR), a vital enzyme in homocysteine metabolism. The patient was commenced on vitamin B12 and folate replacement (with resultant normalization of homocysteine levels) and continued on aspirin. Subsequent visual field testing at two months revealed slight improvement in the visual field defect (ref. Fig. 3) and no further vaso-occlusive events were noted.

## Discussion

Retinal artery occlusion is an indication that the patient is at risk of vascular occlusion elsewhere in the body and therefore requires prompt investigation following diagnosis in the younger population. Ophthalmologists have the opportunity to gain a unique and direct view of a patient’s vascular state. Although ophthalmologists are generally not involved in managing the systemic cause of a patient’s retinal artery occlusion, it is important for ophthalmologists to know about the aetiology and management of retinal artery occlusions in different age groups in order to correctly refer the patient on for optimal and timely management.

Ophthalmological assessment of retinal damage via retinal photography, visual field testing and often fluorescein angiography is warranted [20]. Further systemic investigations include direct visualization of head and neck vasculature via CT angiography/ MRI and carotid duplex ultrasound, an echocardiogram and ECG to identify arrhythmias and structural heart defects that may promote thrombosis/ emboli [20]. A more extensive array of haematological investigations is required in younger patients (< 50 years) to investigate any cause of a ‘hypercoagulable state’ or autoimmune disorder [20]. These include a thrombophilia (coagulation screen, protein C&S, factor V Leiden, anti-phospholipid, plasminogen activator) [1, 2, 20] and autoimmune/vasculitic screen (ANA, anti-double stranded DNA antibody, ANCA, lupus anticoagulant, anticardiolipin antibody) [2, 20]. Other important blood tests include B12 & folate, TFTs, homocysteine levels, blood film, myeloma screen and syphilis screen [1–3].

**Fig. 4:**
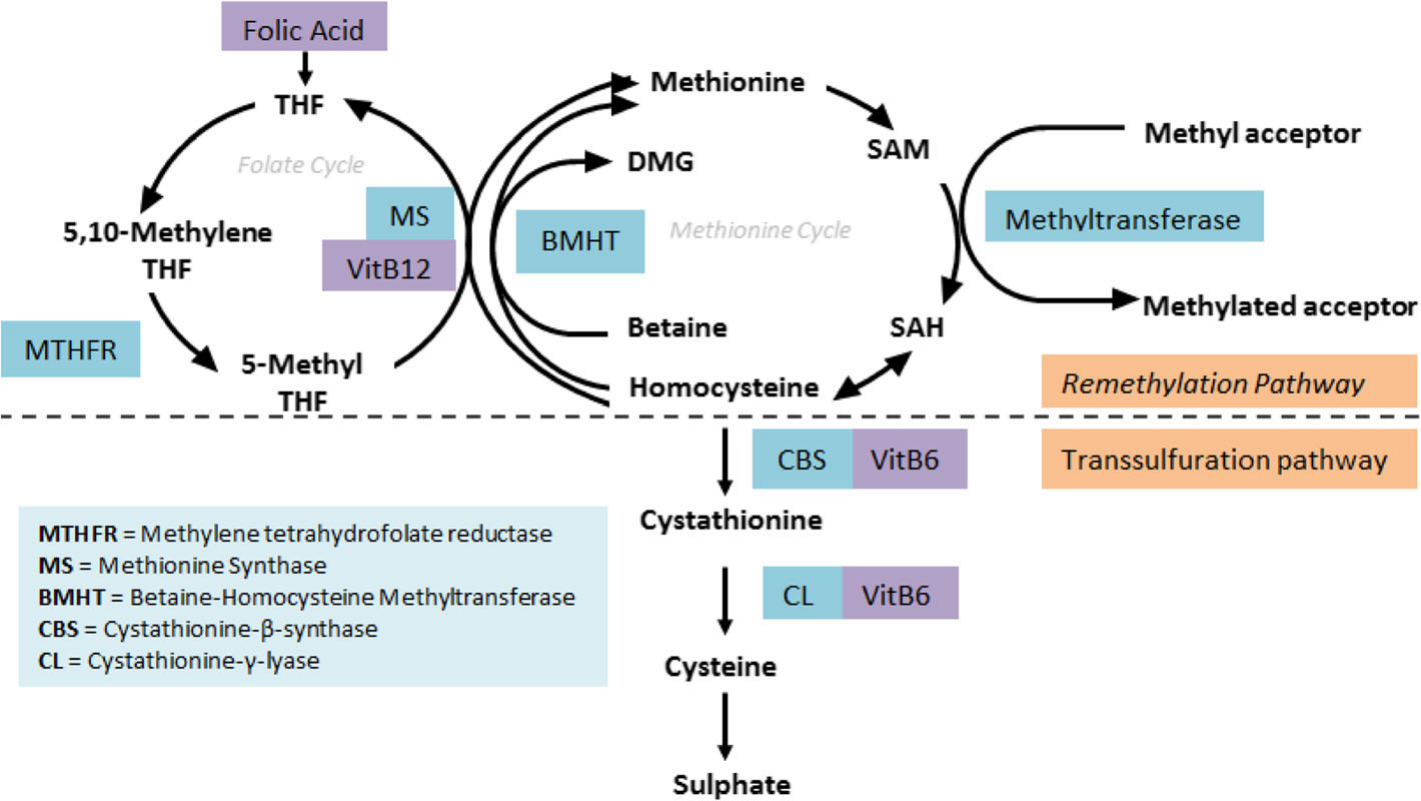
Homocysteine Metabolism

Homocysteine is a sulfhydryl containing amino acid produced as a result of methionine metabolism (an essential amino acid) (ref. Fig. 4) [6]. The two main metabolic pathways are transsulfuration and remethylation, both of which require vital enzymes and vitamin cofactors to function normally. It is therefore understandable that vitamin deficiency and enzymatic defects can result in defective homocysteine metabolism. Auto-oxidated forms of homocysteine are involved in processes that result in increased cell toxicity namely thrombosis, oxidant stress, apoptosis, endothelial cell damage and vascular smooth muscle proliferation [6, 7]. It is via these mechanisms that hyperhomocysteinaemia has been shown to be an independent risk factor for atherosclerotic vascular disease including myocardial infarction, cerebrovascular events and retinal vascular occlusive disease. This risk is graded, relating to an incremental increase in risk per 5 μmol/L increase in homocysteine concentration [8–12].

Hyperhomocysteinaemia is graded as mild (15–30 μmol/L), moderate/intermediate (30–100 μmol/L) and severe (> 100 μmol/L) based on concentrations measured during fasting [6, 21]. Some studies have revealed that between 5 and 10% of the general population have varying levels of hyperhomocysteinaemia and this may reach as high as 30–40% in the elderly population [4, 5].

Homocysteine levels become elevated due to genetic, nutritional and disease related processes, most often occurring in combination. Genetic mutations encoding enzymes involved in homocysteine metabolism include, most commonly, impaired MTHFR enzyme activity which can raise homocysteine levels by up to 25% [6]. The MTHFR enzyme supports conversion of homocysteine to methionine, a vital link in the homocysteine metabolism pathway [22]. The MTHFR gene is located on chromosome 1 with up to 33 rare mutations associated with severe enzyme deficiencies. The C677T (cytosine to thymine mutation at nucleotide 677) genetic defect is a more common mutation, with the homozygous TT mutation being associated with milder enzymatic deficiency [23, 24]. There have been conflicting reports as to whether this genetic mutation in isolation conveys a raised homocysteine to a level where it causes vascular occlusive disease including retinal artery occlusion specifically [6, 25–31]. However, a more dramatic rise in homocysteine levels are seen when this genetic defect occurs in combination with vitamin deficiency [9, 21, 31].

Cystathionine-β-synthase (CBS) mutations result in severely raised homocystiene levels and there are in excess of 100 different types of mutation for this enzyme. The 1278 T subtype is implicated in the rare inherited inborn error of metabolism, homocystinuria [6]. Other clinical features of homocystinuria include mental retardation, skeletal abnormalities, ectopia lentis and congenital glaucoma.

Given that a number of vitamins are important cofactors for homocysteine metabolism, their deficiency can result in accumulation of homocysteine. These dietary vitamins include folate, vitamin B6, and vitamin B12. Even borderline levels of folate deficiency have been associated with raised homocysteine [4]. Folic acid provides a substrate for tetrahydrofolate (THF) within the folate cycle, allowing normal methionine synthase (MS) activity to occur [6]. Vitamin B12 is a vital cofactor in normal MS activity and vitamin B6 is a key factor in normal CBS activity [6].

Chronic disease, namely renal failure, diabetes mellitus, hypothyroidism and severe psoriasis and drugs including anticonvulsants, methotrexate, caffeine, tobacco and alcohol can also contribute to raised homocysteine levels [6, 32].

It makes sense that introducing interventions to lower plasma homocysteine via vitamin replacement should reduce the risk of further vascular occlusive disease and studies have shown that vitamin replacement effectively lowers homocysteine levels [13]. However, meta-analysis of randomized controlled trials looking at homocysteine lowering interventions have failed to associate the use of vitamin replacement with a reduction in major vascular related events [13]. One study specific to young subjects aged between 18 and 40 years revealed that, although the use of B12, folic acid and B6 vitamin replacement reduced the level of homocysteine, it did not cause an improvement in endothelial dependant vasodilatation or antithrombotic function [7].

Also, despite the fact that the relationship between hyperhomocysteinaemia and retinal vascular occlusion has been increasingly reported [14–19], research is yet to be carried out into whether reducing homocysteine levels results in a reduction in the risk of further retinal vascular occlusion [12, 14].

## Conclusions

In this case report, thorough but directed investigation revealed a number of potential causes for this patient’s retinal artery occlusion including migraine (known to cause vasospasm) and a bicuspid aortic valve (with the potential to generate emboli). The leading cause, however, was felt to be the interplay between a diagnosed vitamin B12/folate deficiency and a homozygous MTHFR genetic defect causing high homocysteine levels with resultant premature vessel atherosclerosis.

In summary, branch retinal artery occlusions in the younger population are relatively uncommon but require more extensive investigation of the cause. Hyperhomocysteinaemia promotes accelerated atherosclerosis and is an important risk factor for retinal artery occlusion in young people, as demonstrated in this case. Aetiology of hyperhomocysteinaemia is multifactorial and it is often an interplay between multiple different causes that generates a dangerous level of homocysteine. Although vitamin replacement is often warranted in these cases (including this case), literature does not currently support vitamin replacement as a preventative strategy for further vascular occlusive events. Research is also limited as to whether reducing homocysteine levels lowers the risk of further retinal vascular occlusive events. This therefore represents a potential area for future research and could guide treatment for patients like the one described in this case report. Finally, it is important to note that despite ophthalmology being a highly specialized area of medicine, general medical conditions often manifest with ophthalmological complications where an ophthalmologist will gain the first insight. It is therefore important for ophthalmologists to have knowledge of these conditions allowing for appropriate multidisciplinary team communication and correct ongoing management.

## Abbreviations

ANA: antinuclear antibody
ANCA: Anti-neutrophil cytoplasmic antibody
BMHT: Betaine-homocysteine methyltransferase
BRAO: Branch retinal artery occlusion
CBS: Cystathionine-β-synthase
CL: Cystathionine-γ-lyase
CT: Computed tomography
ECG: Electrocardiogram
MR: Magnetic resonance
MRI: Magnetic resonance imaging
MS: Methionine synthase
MTHFR: Methylenetetrahydrofolate reductase
TFTs: Thyroid function tests
THF: Tetrahydrofolate
